# Supplementary material for: A multiparametric anti-aging CRISPR screen uncovers a role for BAF in protein synthesis regulation
Source: Nat Commun. 2025 Feb 16;16:1681. doi: 10.1038/s41467-025-56916-5 (PMC11830792; doi:10.1038/s41467-025-56916-5)
Supplement: Supplementary file 1 — Supplementary Information [file 41467_2025_56916_MOESM1_ESM.pdf]

Supplementary Information for

# A multiparametric anti-aging CRISPR screen uncovers a role for BAF in protein synthesis regulation

**Authors:**

**Sophia Y. Breusegem[1,6], Jack Houghton[1,7], Raquel Romero-Bueno[2#], Adrián Fragoso-Luna[2#], Katherine A. Kentistou[3], Ken K. Ong[3], Anne F. J. Janssen[1,8], Nicholas A. Bright[1], Christian G. Riedel[4], John R. B. Perry[1,5], Peter Askjaer[2] and Delphine Larrieu[1,9*]**

*Correspondence to: dlarrieu@altoslabs.com

**This PDF file includes:**

Supplementary Figs. 1 to 6

Supplementary Tables 1 to 4

Supplementary Data

Supplementary References

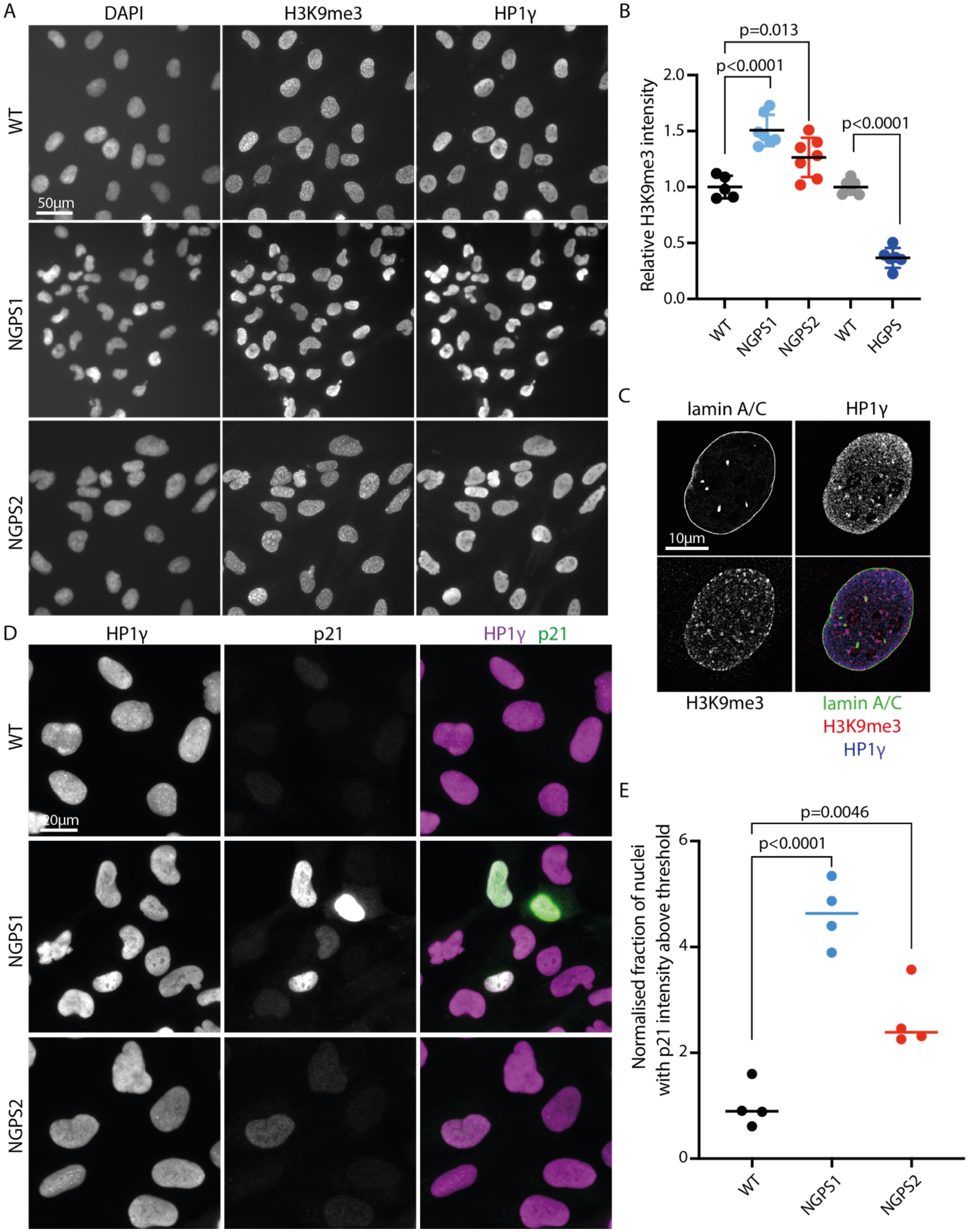


**Supplementary Fig. 1. Heterochromatin marks and p21 are increased in NGPS fibroblasts.**

(**A**) Representative immunofluorescence images of WT, NGPS1 and NGPS2 fibroblasts stained for the heterochromatin marks H3K9me3 and HP1g as indicated. DNA is stained with DAPI. Images were obtained with the high-content microscope. (**B**) Quantification of H3K9me3 intensity in the indicated progeria cell lines, compared to the corresponding WT. Data was obtained using HCS studio and represents the relative expression, in 2 experiments, each averaging 500 nuclei in 3 wells. Lines indicate average ± SD. A one-way ANOVA test with Dunnett's multiple comparisons was used to compare the NGPS cell lines to WT; a student's *t*-test was used to compare HGPS to its corresponding WT (**C**) Representative super-resolution immunofluorescent staining of a WT cell nucleus showing that HP1g overlaps with H3K9me3 and is therefore associated with heterochromatin domains in fibroblasts. (**D**) Representative wide-field immunofluorescence images of HP1g and p21 in WT, NGPS1 and NGPS2 fibroblasts. (**E**) Quantification of the fraction of nuclei showing high level of p21 in the indicated cell lines, compared to the WT. Data was obtained using HCS studio and represents the relative fraction of nuclei with p21 intensity above a set threshold value, as obtained in 4 experiments, each measuring 500 nuclei in 3 wells. Statistical testing used one-way ANOVA with Dunnett's multiple comparisons; lines indicate the median value.

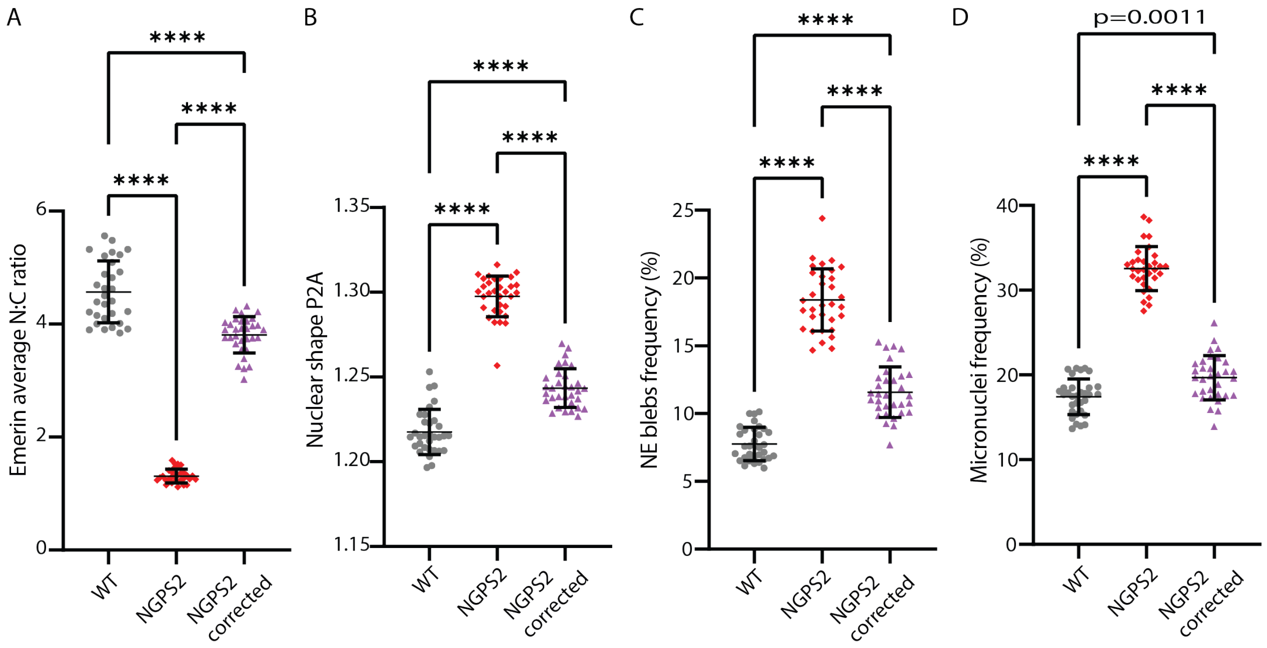


**Supplementary Fig. 2: Validation of the screening phenotypes specificity using NGPS2 corrected isogenic cells.** (**A-D**) Quantification of the indicated phenotype in WT, NGPS2 and NGPS2 corrected cells, in which the BAF A12T mutation has been reversed using CRISPR/Cas9 (*1*). P2A is a perimeter to area (P2A) analysis of nuclear shape. All the data were obtained using HCS Studio software. Each data point is the average value measured over 500 cells, lines indicate the average ± SD. One-way ANOVA was used for statistical analysis using Tukey's multiple comparisons tests. **** indicates $p < 0.0001$.

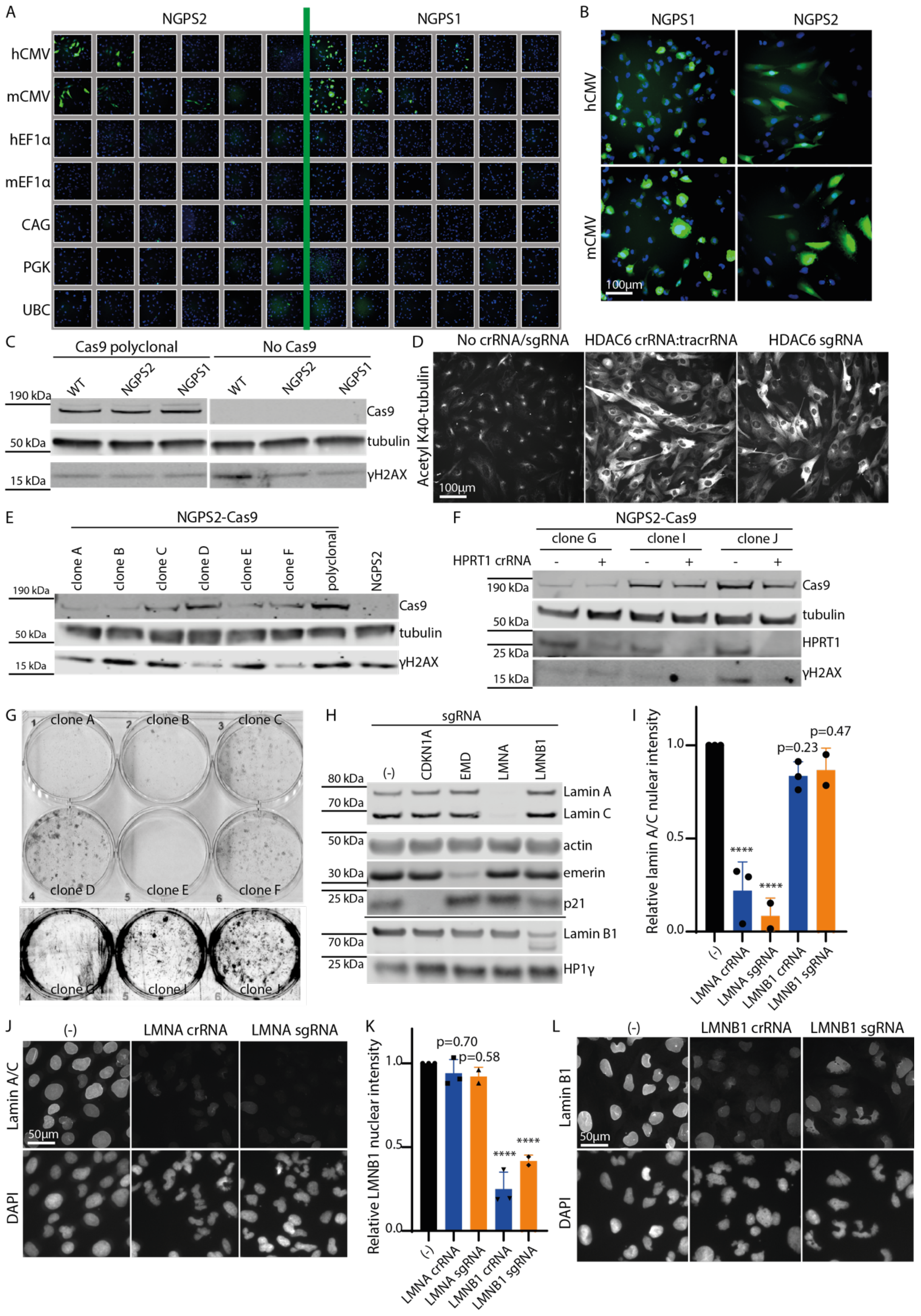
A
NGPS2
NGPS1
hCMV
mCMV
hEF1α
mEF1α
CAG
PGK
UBC
B
NGPS1
NGPS2
hCMV
mCMV
100µm
C
Cas9 polyclonal
No Cas9
WT
NGPS2
NGPS1
190 kDa
50 kDa
15 kDa
Cas9
tubulin
γH2AX
D
No crRNA/sgRNA
HDAC6 crRNA:tracrRNA
HDAC6 sgRNA
Acetyl K40-tubulin
100µm
E
NGPS2-Cas9
clone A
clone B
clone C
clone D
clone E
clone F
polyclonal
NGPS2
F
clone G
clone I
clone J
HPRT1 crRNA
25 kDa
HPRT1
G
H
sgRNA
(-)
CDKN1A
EMD
LMNA
LMNB1
80 kDa
70 kDa
30 kDa
Lamin A
Lamin C
actin
emerin
p21
Lamin B1
HP1γ
I
Relative lamin A/C nuclear intensity
p=0.23
p=0.47
****
LMNA crRNA
LMNA sgRNA
LMNB1 crRNA
LMNB1 sgRNA
J
Lamin A/C
DAPI
50µm
K
Relative LMNB1 nuclear intensity
p=0.70
p=0.58
L
Lamin B1

**Supplementary Fig. 3. Engineering Cas9 expression and assessing its efficiency in NGPS fibroblasts.** (**A**) A GFP construct was transduced in a SMARTchoice promoter selection plate in both NGPS1 and 2 cell lines to identify the promoter leading to the highest protein expression. Nuclei were counterstained with DAPI and the plate was imaged with the high-content microscope. (**B**) Zoom in from images obtained in (A) showing that GFP expression was highest in NGPS fibroblasts with the hCMV promoter. (**C**) Representative western blotting showing that Cas9 expression does not increase gH2AX levels in WT and NGPS polyclonal populations. (**D**) Representative immunofluorescence images showing the increase of acetyl-tubulin on lysine 40 (K40), used as a readout for HDAC6 depletion efficiency following cr:tracr or sgRNA transfection in polyclonal WT-Cas9 cells. The images illustrate efficient but heterogeneous knock-out using both reagents. (**E**) Representative western blot showing the expression level of Cas9 and of the DNA double strand break marker gH2AX in the parental NGPS2 cell line, in the NGPS2-Cas9 polyclonal population or in individual clones grown from single cells. (**F**) Representative western blotting showing the efficiency of HPRT depletion in the indicated NGPS2-Cas9 clones 72 hours post-transfection. (**G**) Resistance to 6-thioguanine was assessed in the indicated clones following HPRT depletion as shown in (F), to identify clones with the highest Cas9 cutting efficiency. (**H**) Representative western blotting showing the efficiency of the indicated sgRNA-mediated gene depletion in NGPS1-Cas9 cells 72 hours post-transfection. (**I-L**) Comparison of the knockout efficiency of the nuclear envelope proteins lamin A/C and lamin B1 (LMNB1) following transfection with a pool of 3 crRNAs or 3 sgRNAs in WT-Cas9 cells and quantified using highcontent microscopy. Representative images of 2-3 independent experiments are shown. Statistical comparison used one-way ANOVA with Dunnett's multiple comparisons testing. **** indicates $p < 0.0001$. Column graphs indicate average ± SD.

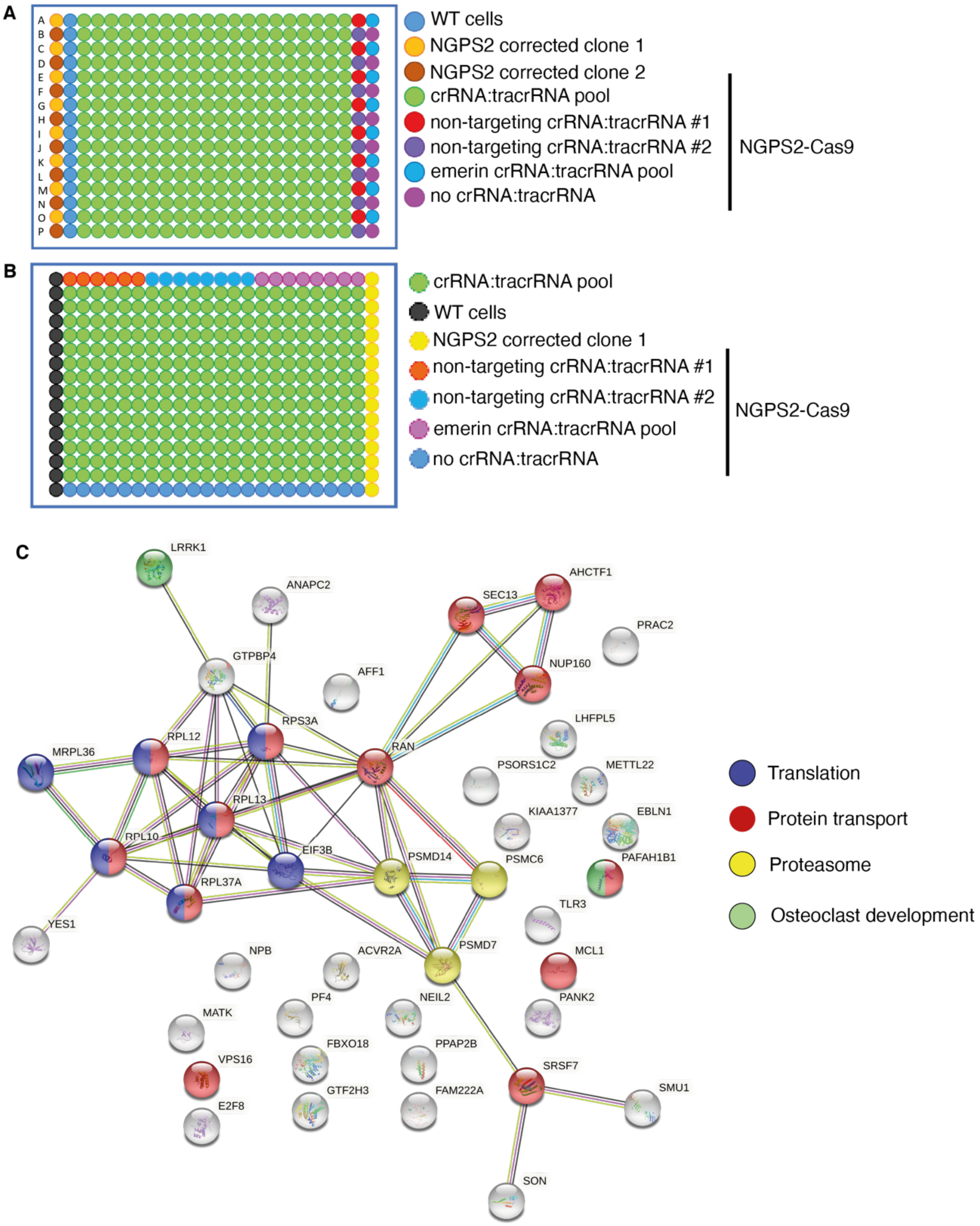


**Supplementary Fig 4. Screening plates layout and validation.** (**A**) 384-well plate layout for the whole genome primary screen. WT cells as well as NGPS2 corrected cells (two separate clones) were used as control cells. Non-targeting crRNAs as well as wells without any crRNA were used

as negative controls for the crRNA and transfection toxicity respectively. Emerin crRNA was used as a positive control for the transfection efficiency on each plate. (**B**) 384-well plate layout for the validation screen. Assay wells now contain a single crRNA:tracrRNA. Controls are as in (A). (**C**) STRING protein-protein interaction diagram. Gene ontology analysis revealed proteins involved in processes of translation (blue nodes), protein transport (red nodes), proteasome (yellow) and osteoclast development (green).

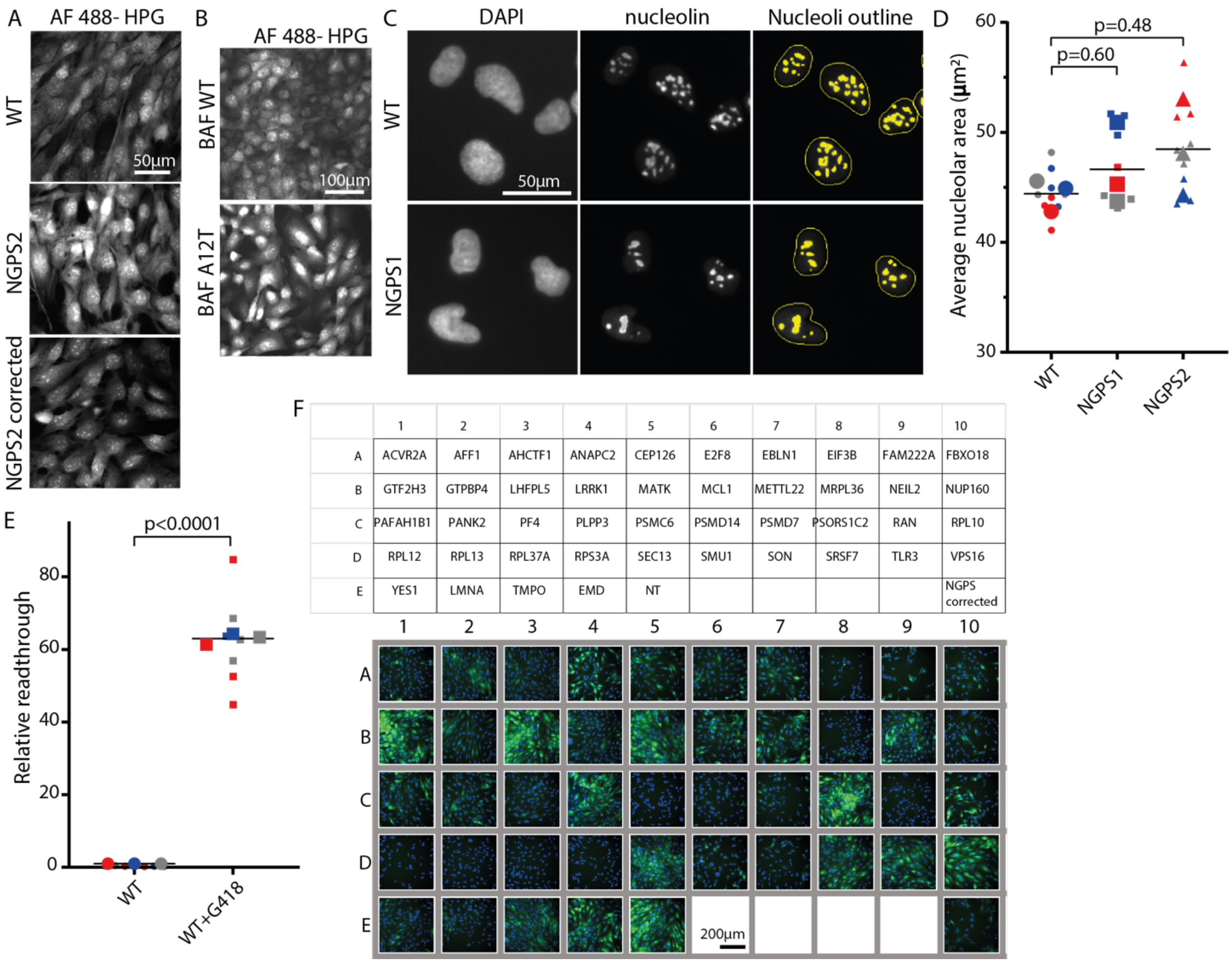


**Supplementary Fig 5. Enhanced protein synthesis in NGPS cells correlates with nuclear envelope abnormalities.** (**A-B**) Nascent protein synthesis assay using HPG incorporation followed by labelling using a "click" reaction with Alexa Fluor 488 azide (AF 488). Representative immunofluorescence images of AF 488 HPG in WT, NGPS2 or NGPS2 corrected (mutation reversed by CRISPR/Cas9) (**A**) or in WT fibroblasts expressing a BAF WT or BAF A12T construct

(**B**). (**C**) Immunofluorescence images of nucleolin in the indicated cell lines used to identify and outline the nucleoli (yellow) using the high-content microscope and analysis software.

DAPI staining was used to define the nuclear mask. (**D**) Average nucleolar area quantified in NGPS1 and NGPS2 compared to WT fibroblasts by high-content microscopy. Separate colors indicate individual experiments. In each experiment 500 nuclei were analyzed in 3 separate wells with the larger symbols indicating the average measured value for each experiment, and lines indicating the average of the averages. Results are compared using one-way ANOVA and Dunnett's multiple comparison testing. (**E**) Translation error rate measured as an increased readthrough using a dual luciferase assay. G418 was used as a positive control for the induction of translational errors *(2)*. Results are derived from the ratio hFluc/hRluc and given as fold induction. Three independent experiments are indicated in a superplot, with separate colors indicating individual experiments and larger symbols indicating the average for each experiment and the line indicating the average of the averages. Results are compared using an unpaired two-tailed *t*-test. (**F**) Top: plate layout indicating the siRNA-targeted genes used in the nascent protein synthesis assay in NGPS2 cells. NT represents a non-targeting siRNA control. Well E10 was seeded with NGPS2 corrected cells as WT control. Bottom: Fluorescence intensity of HPG AF 488 imaged with the high-content microscope showing reduction of protein synthesis in NGPS2 cells upon depletion of most genes, compared to the non-targeting siRNA.

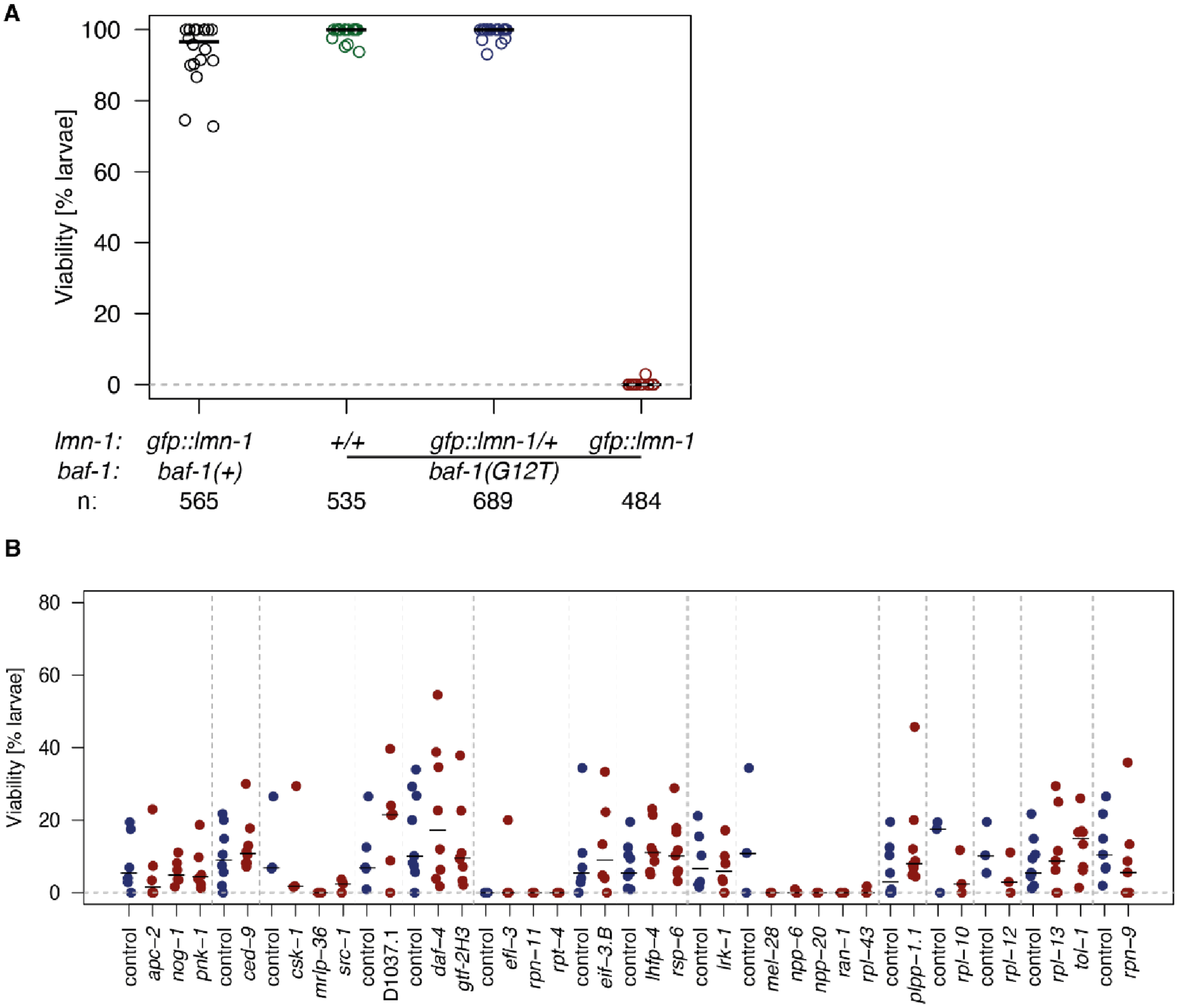


**Supplementary Fig 6. Evaluating the effect of candidate genes knock down in an NGPS *C. elegans* model. (A)** Viability was determined for BAF-1 WT hermaphrodites expressing endogenously tagged GFP::LMN-1 (black circles; strain UD484) and for baf-1(G12T) worms carrying either no gfp::lmn-1 allele (green circles; strain BN808), a single allele (blue circles; strain BN1336) or the gfp::lmn-1 alleles in homozygosity (red circles; strain BN1336). Each circle corresponds to an individual plate and total number of eggs analyzed (n) is indicated. Note that this experiment was performed with *E. coli* OP50 as food source, which induces higher lethality in *gfp::lmn-1; baf-1(G12T)* animals as compared to *E. coli* HT115 used in RNAi experiments. **(B)** Candidate genes were knocked down by RNAi and tested for suppression of lethality in gfp::lmn-

1; baf-1(G12T) hermaphrodites (strain BN1336). Each point corresponds to the percentage of eggs developing into larvae from a single plate with 50-100 eggs laid by 1-day old adult hermaphrodites; 2-13 plates were evaluated in 1-4 experiments for each gene. Black lines indicate medians. Vertical dashed lines separate each set of control and test plates; no significant suppression was observed in any of these cases (Mann-Whitney test).

| Name | Manufacturer | Catalogue number | Application(s) | dilution |
|---|---|---|---|---|
| Anti-emerin | ProteinTech | 10351-1-AP | WB and IF | 1:1000 (WB) 1:750 (IF) |
| Anti-lamin A/C | Santa Cruz Biotechnology | sc-7292 | WB and IF | 1:1000 |
| Anti-b-actin | Cell Signalling | 3700 | WB | 1:4000 |
| Anti-LAP2 | BD Biosciences | 611000 | IF | 1:1000 |
| Anti-a-tubulin | Sigma | T9026 | WB | 1:3000 |
| Anti-lamin B1 | Santa Cruz Biotechnology | sc-365214 | WB and IF | 1:1000 |
| Anti-acetyl-a-tubulin (K40) | Cell Signalling | 5335 | WB and IF | 1:1000 |
| Anti-phospho-histone H2A.X (S139) | Millipore | 05-636-I | WB | 1:200 |
| Anti-53BP1 | Bethyl | A300-272A-M | IF | 1:500 |
| Anti-p21 | Cell Signalling | 2947 | WB and IF | 1:1000 |
| Anti-SIRT7 | Santa Cruz Biotechnology | sc-365344 | WB | 1:200 |
| Anti-RAN | BD Biosciences | 610340 | IF | 1:1000 |
| Anti-HP1g | Santa Cruz Biotechnology | sc-398562 | WB and IF | 1:2000 |
| Anti-H3K9me3 | Abcam | ab8898 | WB and IF | 1:2000 |
| Anti-H3K79me2 | Abcam | ab3594 | WB | 1:1000 |
| Anti-H3 | Cell Signalling | 3638 | WB | 1:1000 |
| Anti-Cas9 | Biolegend | 698301 | WB | 1:1000 |
| Anti-HDAC6 | Abcam | ab133493 | WB | 1:1000 |
| Anti-HPRT1 | Genetex | GTX113466 | WB | 1:1000 |
| Anti-nucleolin | Genetex | GTX13541 | IF | 1:1000 |
| DAPI | EMP Biotech | F-0410-M0001.0-001 | IF | 1:5000 |
| Alexa Fluor 488 antimouse $IgG_{2b}$ | Thermo Fisher Scientific | A21141 | IF | 1:1000 |
| Alexa Fluor 568 antimouse $IgG_1$ | Thermo Fisher Scientific | A21124 | IF | 1:1000 |
| Alexa Fluor 647 antirabbit | Thermo Fisher Scientific | A21241 | IF | 1:1000 |

| | | | | |
|---|---|---|---|---|
| Alexa Fluor 488 azide | Thermo Fisher Scientific | A10266 | IF (Click reaction) | 1:1000 |
| IRDye 680RD anti-rabbit | Licor Biosciences | 925-68073 | WB | 1:15000 |
| IRDye 800CW antimouse | Licor Biosciences | 925-32212 | WB | 1:12000 |
| Prolong Gold | Thermo Fisher Scientific | P10144 | IF | none |

**Supplementary Table 1. List of antibodies and reagents used in Western blotting and immunofluorescence.**

| Catalog number | Target gene | Target Sequence | PAM |
|---|---|---|---|
| CM-007774-01 | SIRT7 | TACCTCCTGCGTTCCCAACA | GGG |
| CM-007774-02 | SIRT7 | GCTGGACCCTAGACACAGGA | TGG |
| CM-003499-01 | HDAC6 | TGCCAGACCCATGAGCAGGT | GGG |
| CM-003499-02 | HDAC6 | CTGCAACTTGTGGGACAGGT | AGG |
| CM-008735-01 | HPRT1 | GATGATCTCTCAACTTTAAC | TGG |
| CM-008735-02 | HPRT1 | GCTTATATCCAACACTTCGT | GGG |

**Supplementary Table 2. crRNA sequences used for CRISPR efficiency testing** (See Figure 2J and Supplementary Figures.3D, 3F). All crRNAs were obtained from Horizon Discovery.

| Gene target | sgRNA sequences |
|---|---|
| AURKA | U*A*A*GUGUUCAUUUAUUGUCC<br>A*U*U*CUGGAAUAUGCACCACU<br>C*C*U*AAUAUUCUUAGACUGUA |
| CDC20 | G*A*A*UGUACUGGCCGUGGCAC<br>U*U*U*GCAAAUGGAGCAGCCUG<br>C*A*C*CUGCACCUCAGCACUGC |
| CDKN1A | U*G*C*AGGCGCCAUGUCAGAAC<br>C*C*A*CUGGGCCGAAGAGGCGG<br>U*C*C*AGGAGGCCCGUGAGCGA |
| CENPE | G*U*U*CAUUAAUACUCACCAAA<br>G*A*U*UUUGGAAAGCAGAGAGA<br>C*A*U*ACCAUCUUUAGGAUGGU |
| EMD | A*A*G*AGAGCUACUUCACCACC<br>A*C*U*GGCGGACAGCCCUGGAC<br>C*C*A*GCCAGCUCACCUGGUGA |
| KIF11 | U*C*U*UAUCAACAGCUCCAGAA<br>U*G*A*CCCUUCCCAAAGUCAAC<br>A*U*C*CUCCAGGAUUCUCUUGG |
| LMNA | C*U*U*UAGCAAUACCAAGAAGG<br>G*G*C*UCUGCUGAACUCCAAGG<br>G*C*A*UGAUCUGCGGGGCCAGG |
| LMNB1 | G*A*C*AGGCGCGUGGGGCUCAG<br>G*C*U*GGCGGUGUACAUCGACA<br>G*C*A*GGUGACGGAGCGCGAGG |
| PLK1 | G*G*C*CCCGCACAUAGCGCCGC<br>C*U*C*GGACGCGGACACCAAGG<br>U*G*C*UGCUCAAGCCGCACCAG |
| SUV39H1 | A*U*C*CAGACUCAGAGAGCACC<br>U*A*U*CCUCAAGCAGUUCCACA<br>G*G*C*CAACUACCUGGUGCAGA |

**Supplementary Table 3. sgRNA sequences used in CRISPR efficiency testing experiments.**

Each gene was targeted by a pool of 3 sgRNA sequences. See Figure 2K-L and Supplementary Fig. 3H-L.

| RNAi | Fw/Rev primers |
|---|---|
| ced-9 | aatactagtATGACACGCTGCACGGCGGACAACTCG/tatctcgagACGTCCATAAGACATTGGACATTGATC |
| csk-1 | aatactagtAAATGTCGGAAATTCAACAGAATACTC/tatctcgagAACCAACTTTATTCTCACTACACAATC |
| daf-4 | aatactagtGATATCAAGTCGAAGAACATTCTTGTG/tatctcgagAATCGGCGGAGGTGGTGGACACGAATC |
| gft-2H3 | aatactagtTATCATTCTGCAATGCTCATCTAGGAC/tatctcgagAGATCCGCCAGTAATATCTGCTGCCTG |
| mrpl-36 | aatactagtaaATGTCCGGAGTATTAAACCGAACTC/tatctcgagAACAAGTCGACAGATTACCAAAGCTTC |
| src-1 | aatactagtAAGCCCAAATAATGAAGCAATGTGATC/tatctcgagTGTCCCTTTGTCATAATCTCATATAAC |

**Supplementary Table 4. PCR primers used in *C. elegans* RNAi experiments**

**Supplementary References:**


1. A. Janssen et al., The BAF A12T mutation disrupts lamin A/C interaction, impairing robust repair of nuclear envelope ruptures in Nestor-Guillermo progeria syndrome cells. Nucleic Acids Res, 50, 9260-9278 (2022).

2. T. Matt et al., Dissociation of antibacterial activity and aminoglycoside ototoxicity in the 4monosubstituted 2-deoxystreptamine apramycin. Proc Natl Acad Sci U S A 109, 10984-10989 (2012).
