# Supplementary material for: A multiparametric anti-aging CRISPR screen uncovers a role for BAF in protein synthesis regulation
Source: Nat Commun. 2025 Feb 16;16:1681. doi: 10.1038/s41467-025-56916-5 (PMC11830792; doi:10.1038/s41467-025-56916-5)
Supplement: Supplementary file 3 — Description of Additional Supplementary Files [file 41467_2025_56916_MOESM3_ESM.pdf]

# <u>Description of Additional Supplementary Files</u>

Supplementary Data 1: Mini library crRNA sequences for CRISPR efficiency testing (Fig. 2L)

Supplementary Data 2: Whole genome primary screen crRNA sequences

Supplementary Data 3: Primary screen results

Supplementary Data 4: Primary screen hits

Supplementary Data 5: Validation screen crRNA sequences

Supplementary Data 6: Validation screen results

Supplementary Data 7: Mini library siRNA sequences for the nascent protein synthesis assay (Fig. 6A)

Supplementary Data 8: Overlap of validated hit genes with human genetic and functional datasets (GWAS)
