# Supplementary material for: A multiparametric anti-aging CRISPR screen uncovers a role for BAF in protein synthesis regulation
Source: Nat Commun. 2025 Feb 16;16:1681. doi: 10.1038/s41467-025-56916-5 (PMC11830792; doi:10.1038/s41467-025-56916-5)
Supplement: Supplementary file 12 — Reporting Summary [file 41467_2025_56916_MOESM12_ESM.pdf]

nature portfolio

Corresponding author(s): Delphine Larrieu

Last updated by author(s): Dec 6, 2024

Reporting Summary

Nature Portfolio wishes to improve the reproducibility of the work that we publish. This form provides structure for consistency and transparency in reporting. For further information on Nature Portfolio policies, see our Editorial Policies and the Editorial Policy Checklist.

Statistics

For all statistical analyses, confirm that the following items are present in the figure legend, table legend, main text, or Methods section.

n/a	Confirmed	
☐	☒	The exact sample size (*n*) for each experimental group/condition, given as a discrete number and unit of measurement
☐	☒	A statement on whether measurements were taken from distinct samples or whether the same sample was measured repeatedly
☐	☒	The statistical test(s) used AND whether they are one- or two-sided<br>*Only common tests should be described solely by name; describe more complex techniques in the Methods section.*
☐	☒	A description of all covariates tested
☐	☒	A description of any assumptions or corrections, such as tests of normality and adjustment for multiple comparisons
☐	☒	A full description of the statistical parameters including central tendency (e.g. means) or other basic estimates (e.g. regression coefficient) AND variation (e.g. standard deviation) or associated estimates of uncertainty (e.g. confidence intervals)
☐	☒	For null hypothesis testing, the test statistic (e.g. *F*, *t*, *r*) with confidence intervals, effect sizes, degrees of freedom and *P* value noted<br>*Give P values as exact values whenever suitable.*
☒	☐	For Bayesian analysis, information on the choice of priors and Markov chain Monte Carlo settings
☒	☐	For hierarchical and complex designs, identification of the appropriate level for tests and full reporting of outcomes
☒	☐	Estimates of effect sizes (e.g. Cohen's *d*, Pearson's *r*), indicating how they were calculated

Our web collection on statistics for biologists contains articles on many of the points above.

Software and code

Policy information about availability of computer code

Data collection	The immunofluorescence images were obtained using an Axioimager Z2 (Zeiss) using ZenBlue 2012 image acquisition software (Zeiss), Hamamatsu Flash 4 sCMOS camera and Zeiss plan-apox lens.<br>In addition, we used an Elyra PS1 (Zeiss) using ZenBlack SR edition image acquisition software (Zeiss), PCO edge 4.2 sCMOS camera and Zeiss plan-apo 63x 1.4 NA lens.<br>For image acquisition and analysis of the high throughput screen, we used a CellInsight CX7 microscope (Thermo) using HCS studio software 2021 (Thermo)
Data analysis	The HCS studio colocalisation bio-application was used to calculate a nuclear shape parameter for the DAPI-defined objects and the emerin intensity in both the nucleus, defined by the DAPI mask, and in a cytoplasmic ring defined by expansion of the DAPI mask. For micronuclei analysis, the LAP2 images were run through the HCS studio micronuclei bio- application. This analysis module was further modified to detect nuclear blebs, again using the LAP2 images . Two multivariate analyses were carried out in R using custom scripts which are described here:<br>Raw genome-wide CRISPR screen data were read into R in a plate-by-plate basis.<br>Prism was used for graph plotting and statistical analysis of data.

For manuscripts utilizing custom algorithms or software that are central to the research but not yet described in published literature, software must be made available to editors and reviewers. We strongly encourage code deposition in a community repository (e.g. GitHub). See the Nature Portfolio guidelines for submitting code & software for further information.

Data

Policy information about availability of data

All manuscripts must include a data availability statement. This statement should provide the following information, where applicable:

- Accession codes, unique identifiers, or web links for publicly available datasets
- A description of any restrictions on data availability
- For clinical datasets or third party data, please ensure that the statement adheres to our policy

The RNA Seq data has been deposited on GEO with the accession number GSE269484: https://www.ncbi.nlm.nih.gov/geo/query/acc.cgi?acc=GSE269484 . eQTL data from the eQTLGen study was accessed using the Phase I release of cis-eQTLs and is available for download via https://eqtlgen.org/phase1.html. GWAS summary statistics are available via https://zenodo.org/records/1251813 from the GIANT consortium, via https://csg.sph.umich.edu/willer/public/glgc-lipids2021/ from the GLGC consortium and via http://www.gefos.org/ from the GEFOS consortium. For the downstream analysis of GWAS data, we used MAGMA (v1.09) and SMR-HEIDI (v0.6886) and no custom code was used. All other source data are provided with the paper.

Research involving human participants, their data, or biological material

Policy information about studies with human participants or human data. See also policy information about sex, gender (identity/presentation), and sexual orientation and race, ethnicity and racism.

Reporting on sex and gender	The NGPS cell lines both come from the only two patients for which cells are available, and both of them are males.
Reporting on race, ethnicity, or other socially relevant groupings	N/A
Population characteristics	N/A
Recruitment	N/A
Ethics oversight	N/A

Note that full information on the approval of the study protocol must also be provided in the manuscript.

Field-specific reporting

Please select the one below that is the best fit for your research. If you are not sure, read the appropriate sections before making your selection.

☒ Life sciences ☐ Behavioural & social sciences ☐ Ecological, evolutionary & environmental sciences

For a reference copy of the document with all sections, see nature.com/documents/nr-reporting-summary-flat.pdf

Life sciences study design

All studies must disclose on these points even when the disclosure is negative.

Sample size	The Sample size is indicated in each Figure legend
Data exclusions	we excluded screening results where <200 cells were detected in the well (toxicity of specific gene KOs)
Replication	Due to the scale of the primary screen, it was only ran once. However, the validation screen was performed in triplicate as detailed in the M&M
Randomization	Clonal populations of C. elegans were used for the study. All nematodes of a particular strain are genetically identical and used in a randomized manner.
Blinding	The bioinformatician who performed the screen data analysis did not know the samples identity

Reporting for specific materials, systems and methods

We require information from authors about some types of materials, experimental systems and methods used in many studies. Here, indicate whether each material, system or method listed is relevant to your study. If you are not sure if a list item applies to your research, read the appropriate section before selecting a response.

Materials & experimental systems

n/a	Involved in the study
☐	☒ Antibodies
☐	☒ Eukaryotic cell lines
☒	☐ Palaeontology and archaeology
☐	☒ Animals and other organisms
☒	☐ Clinical data
☒	☐ Dual use research of concern
☒	☐ Plants

Methods

n/a	Involved in the study
☒	☐ ChIP-seq
☒	☐ Flow cytometry
☒	☐ MRI-based neuroimaging

Antibodies

Antibodies used

Anti-emerin ProteinTech 10351-1-AP WB and IF 1:1000 (WB) 1:750 (IF)
Anti-lamin A/C Santa Cruz Biotechnology sc-7292 WB and IF 1:1000
Anti-b-actin Cell Signalling 3700 WB 1:4000
Anti-LAP2 BD Biosciences 611000 IF 1:1000
Anti-a-tubulin Sigma T9026 WB 1:3000
Anti-lamin B1 Santa Cruz Biotechnology sc-365214 WB and IF 1:1000
Anti-acetyl-a-tubulin (K40) Cell Signalling 5335 WB and IF 1:1000
Anti-phospho-histone H2A.X (S139) Millipore 05-636-I WB 1:200
Anti-53BP1 Bethyl A300-272A-M IF 1:500
Anti-p21 Cell Signalling 2947 WB and IF 1:1000
Anti-SIRT7 Santa Cruz Biotechnology sc-365344 WB 1:200
Anti-RAN BD Biosciences 610340 IF 1:1000
Anti-HP1g Santa Cruz Biotechnology sc-398562 WB and IF 1:2000
Anti-H3K9me3 Abcam ab8898 WB and IF 1:2000
Anti-H3K79me2 Abcam ab3594 WB 1:1000
Anti-H3 Cell Signalling 3638 WB 1:1000
Anti-Cas9 Biolegend 698301 WB 1:1000
Anti-HDAC6 Abcam ab133493 WB 1:1000
Anti-HPRT1 Genetex GTX113466 WB 1:1000
Anti-nucleolin Genetex GTX13541 IF 1:1000
DAPI EMP Biotech F-0410-M0001.0-001 IF 1:5000
Alexa Fluor 488 antimouse IgG2b Thermo Fisher Scientific A21141 IF 1:1000
Alexa Fluor 568 antimouse IgG1 Thermo Fisher Scientific A21124 IF 1:1000
Alexa Fluor 647 antirabbit Thermo Fisher Scientific A21241 IF 1:1000
Alexa Fluor 488 azide Thermo Fisher Scientific A10266 IF (Click reaction) 1:1000
IRDye 680RD anti-rabbit Licor Biosciences 925-68073 WB 1:15000
IRDye 800CW antimouse Licor Biosciences 925-32212 WB 1:12000
Prolong Gold Thermo Fisher Scientific P10144 IF none

Validation

The antibodies used in the screen were thoroughly validated by us using KO, siRNA and overexpression experiments

Eukaryotic cell lines

Policy information about cell lines and Sex and Gender in Research

Cell line source(s)

The NGPS and wild type immortalised fibroblasts were obtained from Carlos Lopez Otin. Both NGPS cell lines come from male patients. Other cell lines were obtained from ATCC or Coriell as described in the manuscript

Authentication

The NGPS cell lines authentication was done via Sanger sequencing of the BANF1 mutation

Mycoplasma contamination

The cells were tested regulary for mycoplasma contamination using Invivogen's mycostrips

Commonly misidentified lines
(See ICLAC register)

No commonly misidentified cell lines were used.

Animals and other research organisms

Policy information about studies involving animals; ARRIVE guidelines recommended for reporting animal research, and Sex and Gender in Research

Laboratory animals

C. elegans - All C. elegans experiments were performed with hermaphrodites.Nucleolar area was measured in 124 nucleoli for the WT worms (strain COP262) and 146 nucleoli for the baf-1(G12T) strain BN1389 1-day old hermaphrodites, representing >20 animals per strain over 4 independent experiments.
For RNAi experiments, 12-15 homozygous yc32[gfp::lmn-1]; baf-1(bq19[G12T]) hermaphrodites (strain BN1336) were used. The

stage of animals imaged/analysed were 1 day old adults and their offspring. Hermaphrodites and males were used to generate new strains via crossing.
For PAFAH1B1, 9 plates were analysed for both the control and PAFAH1B1, over 3 independent experiments. For RPS3A, 15 control plates and 14 RPS3A plates were analysed over 4 independent experiments. For SMU1, 14 control and 16 SMU-1 plates were analysed over 5 independent experiments. For VPS16, 12 controls and 11 VPS16 plates were analysed over 4 independent experiments. For the data presented in Supplementary Fig. 6, hermaphrodites gfp::lmn-1; baf-1(G12T) (strain BN1336) were used. Each point corresponds to the percentage of eggs developing into larvae from a single plate with 50-100 eggs laid by 1-day old adult hermaphrodites; 2-13 plates were evaluated in 1-4 experiments for each gene.

Wild animals

No wild animals were used in this study.

Reporting on sex

Caenorhabditis elegans has two sexes: hermaphrodites and males. The viability experiments reported in Figures 7 and S6 can only be performed with hermaphrodites. There is therefore no sex specific analysis performed.

Field-collected samples

No field-collected samples were used in this study.

Ethics oversight

The use of C. elegans does not require evaluation by ethical committees according to current legislation in the UK and in Europe, as it is a small invertebrate with a simple nervous system

Note that full information on the approval of the study protocol must also be provided in the manuscript.

Plants

Seed stocks

Report on the source of all seed stocks or other plant material used. If applicable, state the seed stock centre and catalogue number. If plant specimens were collected from the field, describe the collection location, date and sampling procedures.

Novel plant genotypes

Describe the methods by which all novel plant genotypes were produced. This includes those generated by transgenic approaches, gene editing, chemical/radiation-based mutagenesis and hybridization. For transgenic lines, describe the transformation method, the number of independent lines analyzed and the generation upon which experiments were performed. For gene-edited lines, describe the editor used, the endogenous sequence targeted for editing, the targeting guide RNA sequence (if applicable) and how the editor was applied.

Authentication

Describe any authentication procedures for each seed stock used or novel genotype generated. Describe any experiments used to assess the effect of a mutation and, where applicable, how potential secondary effects (e.g. second site T-DNA insertions, mosiacism, off-target gene editing) were examined.
